# Association between *TPH1* polymorphisms and the risk of suicide behavior: An updated meta-analysis of 18,398 individuals

**DOI:** 10.3389/fpsyt.2022.932135

**Published:** 2022-07-19

**Authors:** Alma Delia Genis-Mendoza, Yazmín Hernández-Díaz, Thelma Beatriz González-Castro, Carlos Alfonso Tovilla-Zárate, Rosa Giannina Castillo-Avila, María Lilia López-Narváez, Miguel Ángel Ramos-Méndez, Humberto Nicolini

**Affiliations:** ^1^Laboratorio de Genómica de Enfermedades Psiquiátricas y Neurodegenerativas, Instituto Nacional de Medicina Genómica, Ciudad de México, Mexico; ^2^División Académica Multidisciplinaria de Jalpa de Méndez, Universidad Juárez Autónoma de Tabasco, Jalpa de Méndez, Mexico; ^3^División Académica Multidisciplinaria de Comalcalco, Universidad Juárez Autónoma de Tabasco, Comalcalco, Mexico; ^4^División Académica de Ciencias de la Salud, Universidad Juárez Autónoma de Tabasco, Villahermosa, Mexico; ^5^Hospital Chiapas Nos Une “Dr. Gilberto Gómez Maza”, Secretaría de Salud, Tuxtla Gutiérrez, Mexico

**Keywords:** tryptophan hydroxylase, meta-analysis, suicide behavior, polymorphism, risk allele

## Abstract

**Objectives:**

We aimed to examine the association of *TPH1* polymorphisms with the risk of suicide behavior (SB).

**Design:**

Systematic review and meta-analysis.

**Method:**

All relevant studies that evaluated the association between the A218C (rs1800532), A779C (rs1799913) and A6526G (rs4537731) polymorphisms and the susceptibility to SB published up to September 2021 were identified through a comprehensive systematic search in PubMed, Scopus, EBSCO and Science Direct electronic databases. The association between *TPH1* gene polymorphisms and SB was evaluated using inherence models by odds ratio (OR) and 95% confidence interval (CI). Subgroup analyses, heterogeneity analyses, and publication bias were also tested in this meta-analysis.

**Results:**

The meta-analysis for *TPH1* A218C revealed an increased risk of SB in the dominant model (OR = 1.11, 95%CI 1.01–1.22). We also observed a positive association in the allelic (OR = 1.13, 95%CI 1.05–1.21), homozygous (OR = 1.22, 95%CI 1.06–1.40), heterozygous (OR = 1.21, 95%CI 1.08–1.37) and dominant (OR = 1.21, 95%CI 1.09–1.34) inherence models with the suicide attempt. Additionally, in the heterozygous (OR = 0.84, 95%CI 0.73–0.97) and dominant (OR = 0.79, 95%CI 0.68–0.91) inherence models we detected an association with completed suicide. Based on ethnicity, an association of SB in the European population also was observed (OR = 1.29, 95%CI 1.12–1.51). However, for both A779C and A6526G polymorphisms we did not find evidence of an association with SB.

**Conclusion:**

This meta-analysis suggests that the A218C polymorphism of *TPH1* gene could be a possible risk factor of SB. Future large-scale studies are required to analyze the molecular mechanisms by which affect the susceptibility of developing suicide behavior.

## Introduction

Human behavior is governed by a complex interplay of numerous biological, genetic, psychological, cultural, social and family determinants. In particular, suicide behavior (SB) ranges from suicide ideation to suicide attempts and completed suicide and constitutes a multifactorial public health issue. The understanding of the genetic system that causes vulnerability to develop SB is largely incomplete, as its etiology is complex and diverse; however, epidemiological studies show that suicide behavior is partly heritable and polygenic (1, 2).

Serotonin, also known as 5-hydroxytryptamine (5-HT), is produced by generating 5-hydroxytryptophan out of tryptophan and the downstream decarboxylation using aromatic amino acid decarboxylase (3). Tryptophan hydroxylase (TPH) is the rate limiting enzyme in the biosynthesis of serotonin. Serotonin is synthesized in the central nervous system (CNS) as well as gastrointestinal system, and it is produced by TPH, which can be present in two isoforms, TPH1 and TPH2 (4, 5).

The serotonergic system is implicated in the etiology and pathogenesis of several psychiatric disorders. Serotonergic pathways associated with mood disorders and SB include the inflammatory processes related to serotonin processing (6). The neuroimmunological kynurenine pathway has been implicated in major depressive disorder and suicide in adults. Kynurenine is formed from its precursor, tryptophan, if kynurenine levels are associated with tryptophan concentrations, these pathways are argued to have the ability to influence levels of serotonin. It has been reported that the kynurenine production is higher among individuals with a history of suicide attempts (1, 7).

The *TPH1* gene has been extensively studied as a candidate for suicide behavior due to its role in serotonergic neurotransmission (1, 8). This gene is located on chromosome 11p15.3-p14 and has two common polymorphisms in intron 7 consisting of A for C substitution at nucleotides 779 (A779C; rs1799913) and at 218 (A218C; rs1800532), plus one polymorphism in the promoter region consisting of A for G substitution at nucleotides 6526 (A6526G; rs4537731). These polymorphisms have been associated with suicide for they may influence the level of serotonin metabolites and their functionality (9, 10).

Although one meta-analysis (10) has already addressed the issue of the association between the *TPH1* polymorphisms and (11) SB, the sample sizes used were relatively small. Also, in the las years, the *TPH1* polymorphisms have been attracting attention and more studies have explored the association of *TPH1* polymorphisms and suicide behavior. Therefore, it is important to summarize the results from more studies to further validate the association of *TPH1* polymorphisms with the risk of suicide behavior. In this study, a systematic review and updated meta-analysis was performed on all eligible case-control studies to estimate the overall SB risk associated with three polymorphisms of *TPH1* gene: A218C, A779C and A6526G. Additionally, we conducted subgroup analyses stratified by ethnicity and diagnostic. The results of this meta-analysis can provide an opportunity to unveil the role that the *TPH1* gene plays in the susceptibility to suicide.

## Materials and methods

The search strategy follows the PRISMA (http://www.prisma-statement.org/) reporting guidelines.

### Search strategy

**Figure 1:**
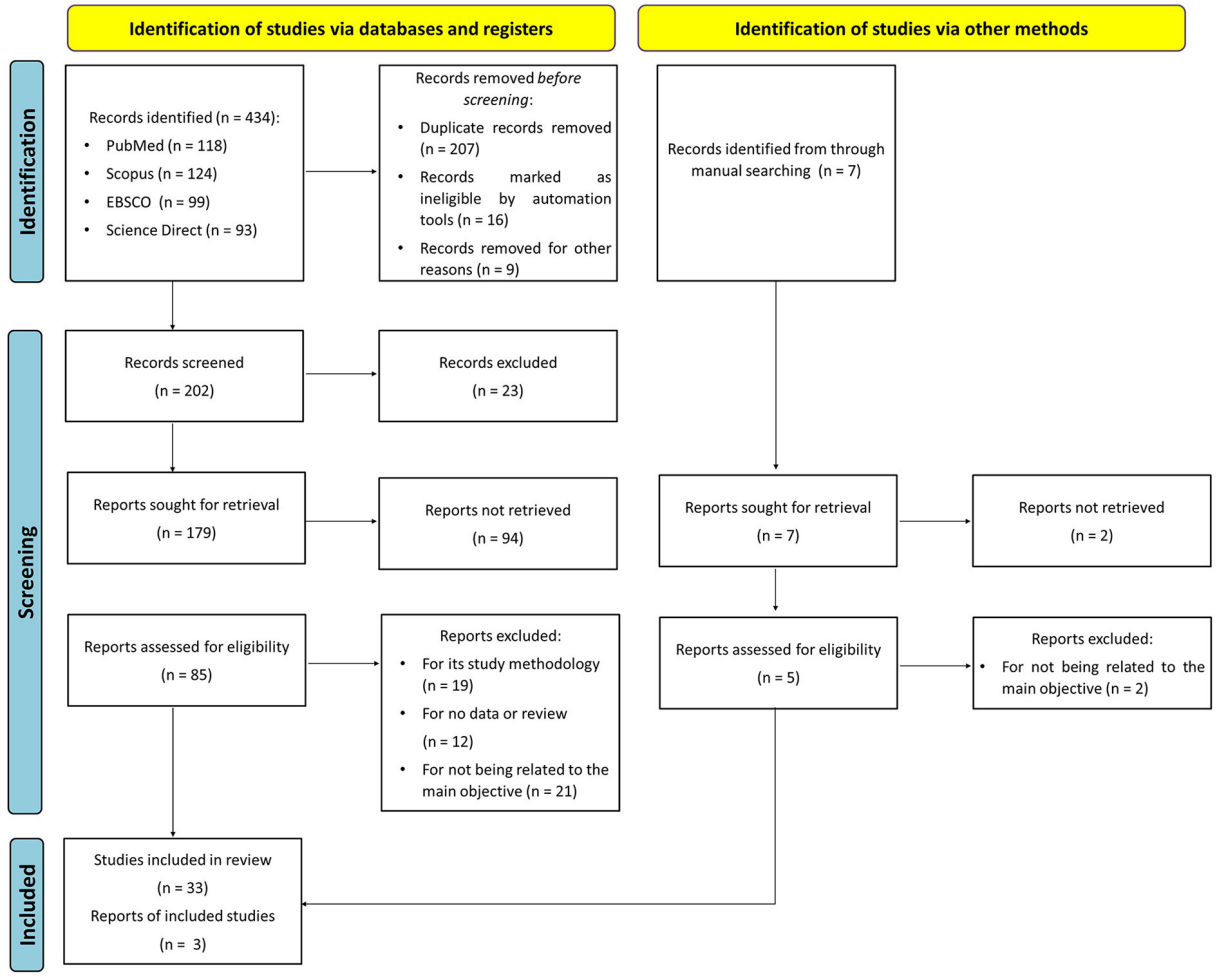
The flow diagram of study selection.

The following terms were used for searching potential studies: (“suicide” OR “suicidal”) AND (“TPH1” OR “tryptophan hydroxylase 1”). We searched for articles were searched in PubMed, Scopus, EBSCO and Science Direct databases, dated up to September 2021. To find other potential studies, we examined the references of the eligible studies. The process of the study selection in the preset work is detailed in a flow diagram; Figure 1.

### Eligible inclusion/exclusion criteria

The criteria applied for eligible studies were: 1- studies that evaluated the association between *TPH1* A218C, A779C and A6526G polymorphisms and suicide risk, 2- cases-controls designs studies, 3- individuals with a suicidal behavior which identified by a specialist; we included all the suicidal behavior spectrum (attempt, ideation and completed suicide) addressed in the published studies, 4- sufficient data available to obtain genotypic frequencies to calculate odds ratio (OR) and 95% confidence interval (95%CI), 5- articles published in English. These data were not always available for all studies, but when needed, we contacted the authors to clarify the information not included in the papers. Reviews, meta-analysis, duplicates, case reports, book chapters, and animal studies were all excluded.

### Data collection

The relevant data from each study were extracted by to reviewers (Hernandez-Díaz and Castillo-Avila) using standardized and structured forms. The following data were extracted: first author, year of publication, country, main outcome, criteria diagnostic, source of DNA, genotyping method, ethnicity, gender, sample size, genotype distribution for the cases and controls. Once encountering discrepancies, other authors re-checked the original articles until an agreement was achieved by all of them.

### Assessment of methodological quality

The Newcastle-Ottawa Scale (NOS) was used to examine the quality of each study. Quality scores range from 0 to 9, and higher scores mean better quality of the study. Studies scoring six o higher were considered as high-quality studies. The qualities of the includes studies were evaluated by the same two investigators. Disagreements were resolved through discussion by a third investigator (Gonzalez-Castro).

### Statistical analysis

The relationship between *TPH1* gene polymorphism and SB risk was determined by calculating OR and 95%CI. To measure this association, we evaluated the effect by five genetic models: allelic (A vs. C), dominant (AA + AC vs. CC), recessive (AA vs. AC + CC), heterozygous (AC vs. CC) and homozygous (AA vs. CC).

Z-test was used to determine the significance of the OR (*P* < 0.05 was considered statistically significant). Cochrane *Q*-test and I^2^ test was conducted to assess the between studies heterogeneity. Significant heterogeneity was defined with a *P*-value < 0.05 and *I*^2^ ≥ 50% and a random effect model (DerSimonian–Laird) was used in this case. Otherwise, a fixed effect model (Mantel–Haenszel) was applied. Subgroup analysis was used to further identify the factors influencing heterogeneity. Publication bias was assessed by funnel diagram that produces a diagram according to its OR and the standard error of each study, moreover, Egger's test was calculated (*P* < 0.05 was considered statistically significant). The data were analyzed using Comprehensive Meta-Analysis (CMA) software v.2.

## Results

### Literature search and study characteristics

A total of 441 potentially relevant studies emerged from the first search in PubMed, EBSCO, Science Direct, Scopus and manual searching. After excluding 351 unrelated publications, we screened 90 publications for eligibility of inclusion. Finally, thirty-six studies met the selection criteria and were finally enrolled for pooled analyses (11–47). The PRISMA flow diagram used for study selection process is summarized in Figure 1.

There were 6,214 cases and 12,184 controls in these 36 studies, 16 studies were conducted in European populations, 13 in Asian populations, 5 in American populations, 1 in Turkish and 1 in mixed populations (USA and Italy). In terms of the SB type, some studies evaluated suicide attempt (*n* = 27), suicide ideation (*n* = 1), and completed suicide (*n* = 8). The age of the individuals studied ranged from 17.4 to 65 years old. There were statistically significant deviations from the HWE in the control groups (*n* = 3); therefore, these studies were excluded from the meta-analysis.

According to the Newcastle–Ottawa scale, the quality score of each included study was >6, indicating good quality overall. Tables 1, 2 displays the main characteristics and quality assessment results of the eligible studies.

**Table 1:** The characteristics of included studies in this meta-analysis for A218C polymorphism.

**References**	**Country**	**Sample**		**Males**		**Mean age**		**SB**	**Genotypes**						**Alleles**				**P for HWE**		**NOS scale**
		**Case**	**Control**	**Case**	**Control**	**Case**	**Control**		**Case**			**Control**			**Case**		**Control**		**Case**	**Control**	
									**AA**	**AC**	**CC**	**AA**	**AC**	**CC**	**A**	**C**	**A**	**C**			
*A218C*																					
Bellivier F (12)	France	52	94	-	57	-	43.7	SA	17	24	11	11	45	38	58	46	67	121	0.642	0.672	6
Kunugi H (14)	Japan	46	208		95	55	32.1	SA	10	29	7	55	105	48	49	43	215	201	0.07	0.876	7
Geijer T (19)	Sweden	165	98	57	64	41	38	SA	30	87	48	13	47	38	147	183	73	123	0.387	0.797	8
Paik I (21)	Korea	27	236		124	30	29.6	SA	4	12	11	66	116	54	20	34	248	224	0.806	0.824	7
Ono H (20)	Japan	132	132	90	90	48.3	47.2	CS	29	68	35	26	71	35	126	138	123	141	0.709	0.353	8
Souery D (25)	Belgium, Bulgaria, Croatia, Germany, Greece, Israel, Italy, Sweden, United Kingdom	167	167	-	-	-	-	SA	29	85	53	27	74	66	143	191	128	206	0.61	0.418	6
Abbar M (22)	France	231	281	105	170	36	43	SA	43	120	68	30	133	118	206	256	193	369	0.435	0.406	8
Zalsman G (27)	Israel	84	172	40	74	17.4	40.9	SA	16	34	34	41	85	46	66	102	167	178	0.164	0.887	8
Turecki G (26)	Canada	101	129	-	-	32.2	34	CS	18	48	35	18	71	40	84	118	107	151	0.826	0.128	6
Hong CJ (23)	Taiwan	140	251	-	123	35.3	33.8	SA	42	57	41	42	135	74	141	139	219	283	0.028	0.138	7
Rujescu D (28)	Germany	86	154	27	66	39	48	SA	10	48	28	19	78	57	68	104	116	192	0.12	0.328	8
Rujescu D (30)	Germany	147	326	52	-	40	-	SA	18	81	48	40	155	131	117	177	235	417	0.069	0.572	7
Jernej B (31)	Croatia	185	358	144	298	52	43	CS	84	85	16	202	141	15	253	117	545	171	0.395	0.115	8
Ohtani M (32)	Japan	132	214	94	114	55.4	63.5	CS	30	60	42	44	113	57	120	144	201	227	0.338	0.38	8
Stefulj J (34)	Croatia	160	284	160	284	48	44	CS	21	69	70	44	145	95	111	209	233	335	0.541	0.352	8
Stefulj J (36)	Croatia	247	320	247	320	65	65	CS	33	111	103	50	162	108	177	317	262	378	0.72	0.401	8
Viana MM (37)	Brazil	248	63	142	36	37.2	34.3	SA	45	125	78	7	31	25	215	281	45	81	0.679	0.569	9
Liu X (35)	China	297	174	171	98	46.1	42.8	SA	72	148	67	41	84	49	292	282	166	182	0.591	0.668	9
Yoon H K (38)	Korea	191	193	70	73	40.5	41.4	SA	49	97	45	60	85	48	195	187	205	181	0.823	0.107	8
Baud P (39)	Switzerland and France	544	1,027	166	-	39.1	-	SA	99	256	182	181	483	363	454	620	845	1,209	0.59	0.354	6
Wilson ST (40)	USA	71	101	-	39	34.2	40.5	SA	13	44	14	22	43	36	70	72	87	115	0.043	0.185	7
Buttenschon HN (42)	Denmark	490	1,027	209	763	-	-	CS	80	228	182	181	483	363	388	592	845	1,209	0.546	0.354	6
Beden O (43)	Turkish	109	98	36	50	33.6	36.5	SA	24	53	32	14	42	42	101	117	70	126	0.816	0.509	8
Lee SM (46)	Korea	13	20	1	7	31.9	33.6	SA	1	8	4	7	10	3	10	16	24	16	0.279	0.852	8
Choi HY (45)	Korea	71	89	36	41	38.5	41.1	SI	14	46	11	20	46	22	74	68	86	90	0.012	0.666	8
Choi HY (45)	Korea	69	143	34	65	39.3	44.9	SA	13	42	13	32	74	37	68	68	138	148	0.052	0.664	8
Pan YF (47)	China	712	739	235	247	40.5	40.2	SA	156	362	194	156	387	196	674	750	699	779	0.598	0.17	9
Total		4,917	7,098																		

*SA, suicide attempt, SI, suicide ideation, CS, completed suicide*.

**Table 2:** The characteristics of included studies in this meta-analysis for A779C and A6528G polymorphisms.

**References**	**Country**	**Sample**		**Males**		**Mean age**		**SB**	**Genotypes**						**Alleles**				**P for HWE**		**NOS scale**
		**Case**	**Control**	**Case**	**Control**	**Case**	**Control**		**Case**			**Control**			**Case**		**Control**		**Case**	**Control**	
									**AA**	**AC**	**CC**	**AA**	**AC**	**CC**	**A**	**C**	**A**	**C**			
*A779C*																					
Nielsen DA (11)	USA	102	232	-	232	31	32	SA	17	57	28	49	106	77	91	113	204	260	0.186	0.268	7
Rotondo A (15)	Italy and USA	97	153	-	153	32	32	SA	12	50	35	33	68	52	74	120	215	201	0.363	0.229	7
Kunugi H (14)	Japan	46	208	7	95	55	32	SA	10	29	7	55	105	48	49	43	215	201	0.07	0.876	7
Roy A (24)	Sweden	24	158	15	77	-	-	SA	2	12	10	35	86	37	16	32	156	160	0.54	0.264	6
Rujescu D (28)	Germany	86	154	27	66	39	48	SA	10	47	29	20	76	58	67	105	116	192	0.166	0.526	8
Rujescu D (30)	Germany	147	326	52	148	40	46	SA	48	81	18	131	155	40	177	117	417	235	0.069	0.572	7
Pooley EC (29)	United Kingdom	129	329	52	138	38	38	SA	20	67	42	44	135	150	107	151	223	435	0.427	0.126	9
Ohtani M (32)	Japan	134	325	95	171	55.4	63.5	CS	30	59	42	45	138	82	119	143	228	302	0.294	0.311	9
Koller G (33)	Germany	80	241	-	79	27.3	42.6	SA	15	38	27	36	114	91	68	92	186	296	0.801	0.975	7
Liu X (35)	China	266	164	171	98	46.1	42.8	SA	77	126	63	43	85	36	280	252	171	157	0.414	0.622	9
Pompili M (44)	Roma	57	54	36	34	48.6	51.4	SA	8	25	21	13	27	17	41	67	53	61	0.899	0.717	8
Lee SM (46)	Korea	13	20	1	7	31.9	33.6	SA	1	8	4	8	9	3	10	16	25	15	0.279	0.858	8
Pan YF (47)	China	712	739	235	247	40.5	40.2	SA	158	361	193	163	386	190	677	747	712	766	0.659	0.21	9
Total		1,893	3,103																		
*A6526G*																					
Ono H (20)	Japan	132	132	90	90	48.3	47.2	CS	89	42	1	89	38	5	220	44	216	48	0.094	0.709	8
Turecki G (26)	Canada	101	129	-	-	32.2	34	CS	35	46	20	45	62	22	116	86	152	106	0.49	0.934	6
Liu X (35)	China	283	180	171	98	46.1	42.8	SA	163	96	24	87	77	16	422	144	251	109	0.074	0.859	9
Total		516	441																		
*Systematic review*																					
*A218C*																					
Tsai SJ (16)	China	41	200	-	91	-	-	SA	17	15	9	33	113	54	49	33	179	221	0.125	0.043	6
Du L (18)	Hungary	35	84	27	60	47.7	56.6	CS	6	24	5	13	52	19	36	34	78	90	0.027	0.025	8
Saetre P (41)	Denmark, Norway and Sweden	299	1,655	-	845	45	44	SA	99	150	50	570	391	694	348	250	1,531	1,779	0.591	0	7
Total		375	1,939																		
*A779C*																					
Saetre P (41)	Denmark, Norway and Sweden	299	1,655	-	845	45	44	SA	99	150	49	570	391	694	346	250	1,531	1,779	0.535	0	7
Total		674	3,594																		

### *TPH1* A218C polymorphism and the risk of SB

**Figure 2:**
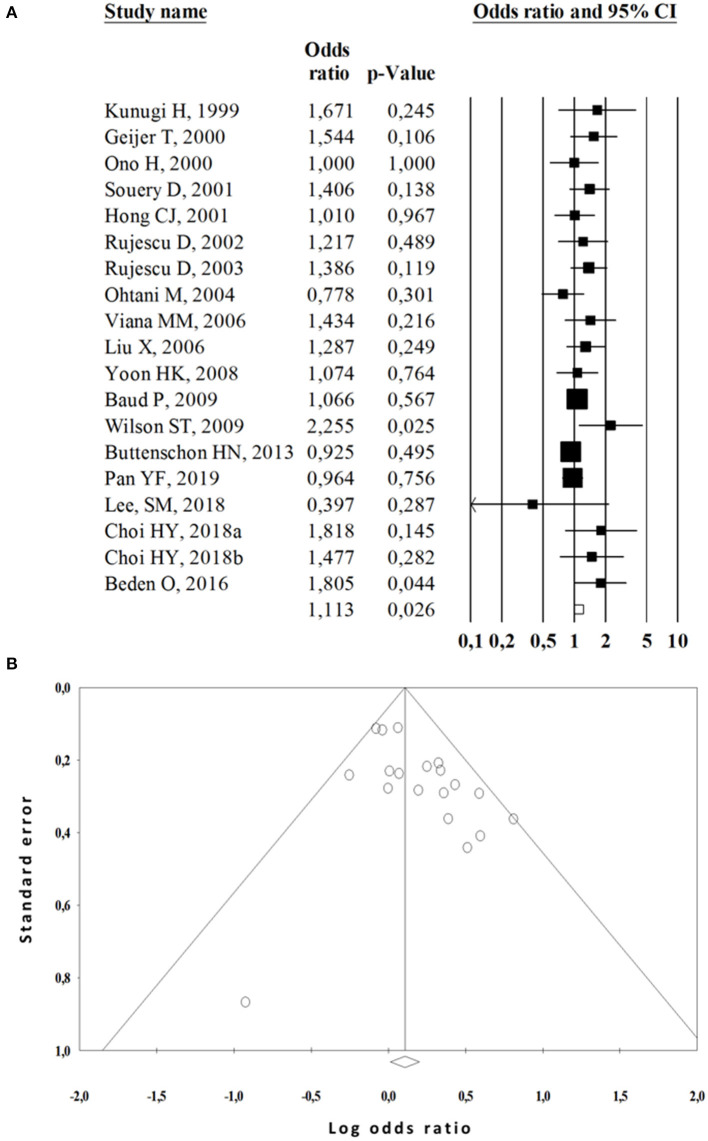
Meta-analysis of the association between A218C polymorphism and suicide behavior risk. **(A)** Forest plot of overall analysis in dominant comparison. **(B)** Funnel plot of overall analysis in dominant comparison.

Twenty-seven studies (4,917cases and 7,098 controls) assessed the relationship between *TPH1* A218C polymorphism and the risk of suicide behavior (12, 14, 19–43, 45–47). The integrated analyses demonstrated that the AA/AC genotypes of A218C polymorphism was significantly associated with an increased risk of SB compared with the CC genotype (OR = 1.11, 95%CI 1.01–1.22; *P* = 0.026, Q test = 0.212, *I*^2^ = 19.90) (Figure 2A).

**Figure 3:**
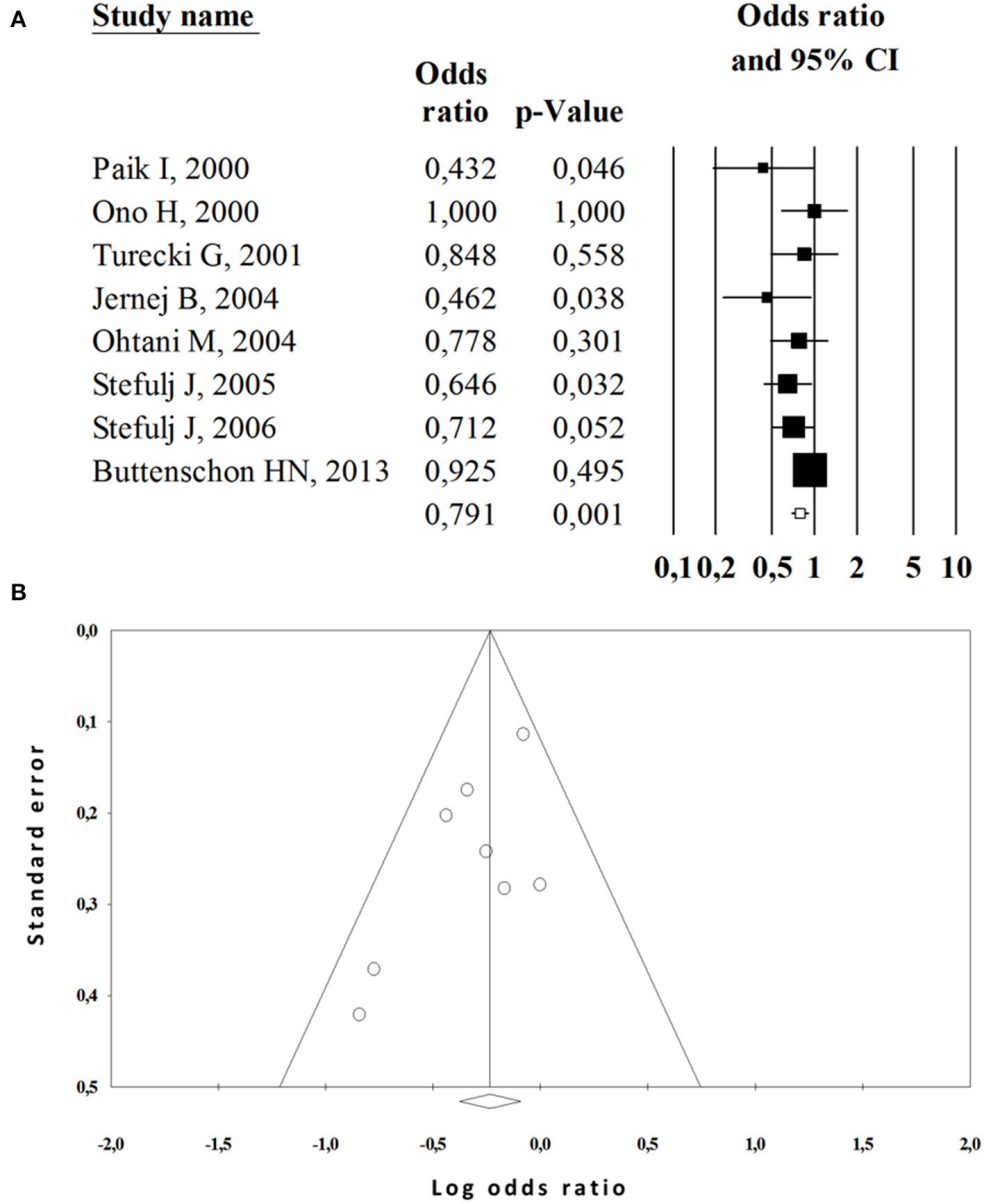
Meta-analysis of the association between A218C polymorphism and completed suicide risk. **(A)** Forest plot of subgroup analysis in dominant comparison. **(B)** Funnel plot of subgroup analysis in dominant comparison.

Subgroup analyses were then performed based on ethnicity and diagnostic in order to investigate sources of heterogeneity (Table 3). We observed a positive association in the allelic (OR = 1.13, 95%CI 1.05–1.21; *P* = 0.000, Q test = 0.233, *I*^2^ = 19.72), homozygous (OR = 1.22, 95%CI 1.06–1.40; *n* = 0.004, Q test = 0.117, *I*^2^ = 30.77), heterozygous (OR = 1.21, 95%CI 1.08–1.37; *P* = 0.001, Q test = 0.186, *I*^2^ = 23.60) and dominant (OR = 1.21, 95%CI 1.09–1.34; *P* = 0.000, Q test = 0.175, *I*^2^ = 24.71) inherence models with the suicide attempt; while a decreased risk of completed suicide was observed in the heterozygous model (OR = 0.84, 95%CI 0.73–0.97; *P* = 0.023, Q test = 0.153, *I*^2^ = 33.09) and dominant model (OR = 0.79, 95%CI 0.68–0.91; *P* = 0.001, Q test = 0.317, *I*^2^ = 14.38) (Figure 3A). In the subgroup analysis based on ethnicity, A218C was associated with increased SB risk in European populations according to the dominant model (OR = 1.29, 95%CI 1.12–1.51; *P* = 0.001, Q test = 0.117, *I*^2^ = 43.22). However, no significant associations were found in Asian populations.

**Table 3:** Integrated analyses for *TPH1* gene polymorphisms and suicide behavior.

**Model**	**OR (CI 95%)**	***Z P*-value**	***Q*-test *P*-value**	*I* ^2^	**Egger's test *P*-value**
***A218C***					
**Overall**					
A vs. C	1.02 (0.96–1.08)	0.456	0.175	22.29	0.343
AA vs. CC	1.01 (0.89–1.13)	0.875	0.109	27.65	0.989
AC vs. CC	1.03 (0.94–1.13)	0.482	0.122	27.29	0.191
AA vs. AC+CC	0.98 (0.88–1.08)	0.690	0.281	13.17	0.807
AA+AC vs. CC	1.11 (1.01–1.22)	**0.026**	0.212	19.90	0.212
**Suicide attempt**					
A vs. C	1.13 (1.05–1.21)	**0.000**	0.233	19.72	0.444
AA vs. CC	1.22 (1.06–1.40)	**0.004**	0.117	30.77	0.199
AC vs. CC	1.21 (1.08–1.37)	**0.001**	0.186	23.60	0.228
AA vs. AC+CC	1.11 (0.98–1.25)	0.091	0.128	29.49	0.765
AA+AC vs. CC	1.21 (1.09–1.34)	0.000	0.175	24.71	0.145
**Competed suicide**					
A vs. C	0.92 (0.81–1.04)	0.224	0.032	49.30	0.995
AA vs. CC	0.86 (0.72–1.03)	0.121	0.157	30.43	0.784
AC vs. CC	0.84 (0.73–0.97)	**0.023**	0.153	33.09	0.253
AA vs. AC+CC	0.88 (0.76–1.03)	0.114	0.594	0.000	0.664
AA+AC vs. CC	0.79 (0.68–0.91	**0.001**	0.317	14.38	0.179
**European population**					
A vs. C	0.96 (0.88–1.05)	0.392	0.146	38.99	0.702
AA vs. CC	1.00 (0.83–1.20)	0.975	0.345	11.04	0.620
AC vs. CC	1.12 (0.98–1.28)	0.084	0.102	41.52	0.372
AA vs. AC+CC	1.01 (0.86–1.18)	0.846	0.118	39.17	0.284
AA+AC vs. CC	1.29 (1.12–1.51)	**0.001**	0.117	43.22	0.107
**Asian population**					
A vs. C	1.01 (0.93–1.11)	0.674	0.256	19.71	0.272
AA vs. CC	1.06 (0.88–1.28)	0.494	0.260	19.23	0.326
AC vs. CC	1.00 (0.86–1.17)	0.957	0.189	26.75	0.595
AA vs. AC+CC	1.01 (0.81–1.26)	0.924	0.082	40.02	0.170
AA+AC vs. CC	1.00 (0.86–1.15)	0.998	0.344	10.53	0.856
*A779C*					
**Overall**					
A vs. C	0.98 (0.89–1.08)	0.738	0.552	0.000	0.337
AA vs. CC	0.97 (0.81–1.16)	0.778	0.224	21.71	0.115
AC vs. CC	1.05 (0.91–1.21)	0.484	0.256	18.58	0.842
AA vs. AC+CC	0.89 (0.77–1.04)	0.152	0.216	22.46	0.651
AA+AC vs. CC	1.02 (0.89–1.17)	0.686	0.202	23.90	0.637
**Suicide attempt**					
A vs. C	0.92 (0.79–1.07)	0.326	0.009	56.12	0.205
AA vs. CC	0.94 (0.78–1.14)	0.569	0.216	23.20	0.114
AC vs. CC	1.07 (0.92–1.24)	0.337	0.246	20.07	0.833
AA vs. AC+CC	0.90 (0.77–1.05)	0.210	0.250	19.71	0.134
AA+AC vs. CC	1.03 (0.90–1.19)	0.620	0.155	29.65	0.654
**European population**					
A vs. C	0.94 (0.79–1.13)	0.566	0.336	11.36	0.857
AA vs. CC	1.01 (0.73–1.41)	0.925	0.121	42.65	0.105
AC vs. CC	1.20 (0.94–1.54)	0.140	0.195	32.11	0.109
AA vs. AC+CC	0.86 (0.66–1.12)	0.286	0.330	13.26	0.593
AA+AC vs. CC	1.23 (0.96–1.57)	0.088	0.259	24.31	0.107
**Asian population**					
A vs. C	0.99 (0.88–1.11)	0.917	0.382	4.411	0.475
AA vs. CC	1.00 (0.79–1.27)	0.951	0.366	7.189	0.552
AC vs. CC	0.92 (0.75–1.12)	0.412	0.575	0.000	0.699
AA vs. AC+CC	1.05 (0.86–1.27)	0.606	0.208	32.02	0.388
AA+AC vs. CC	0.94 (0.78–1.13)	0.549	0.608	0.000	0.998
*A6526G*					
**Overall**					
A vs. G	1.12 (0.91–1.38)	0.247	0.458	0.000	0.583
AA vs. GG	1.14 (0.69–1.86)	0.602	0.298	17.34	0.374
AG vs. GG	0.90 (0.55–1.47)	0.689	0.252	27.42	0.128
AA vs. AG+GG	1.20 (0.92–1.56)	0.175	0.378	0.000	0.108
AA+AG vs. GG	1.01 (0.63–1.60)	0.955	0.284	20.64	0.207

### *TPH1* A779C polymorphism and the risk of SB

Thirteen studies (1,893 cases and 3,103 controls) assessed relationship between *TPH1* A779C polymorphism and the risk of suicide behavior (11, 14, 15, 24, 28–30, 32, 33, 35, 44, 46, 47). Overall, we did not observe an association between A779C polymorphisms of *TPH1* gene in the allele frequencies or any genotype model with an overall SB risk (Table 3). Either in a subgroup analysis by ethnicity or by diagnostic, no significant SB risk was identified.

### *TPH1* A6526G polymorphism and the risk of SB

Three case–control studies with 516 patients and 441 controls were included in the present meta-analysis (20, 26, 35). The major outcomes in this study are summarized in Table 3. The *TPH1* gene A6526G polymorphism was not significantly associated with suicide behavior under any genetic model.

### Publication bias and sensitivity analysis

We estimated potential publication bias in this meta-analysis with funnel plots and Egger's test. Funnel plots were found to be overall symmetrical (Figures 2B, 3B) and the P values for Egger's test were >0.05 in all comparisons (Table 3). These results indicated that our quantitative pooled analysis results were not likely to be seriously influenced by publication biases. We carried out a sensitivity analysis to evaluate the influence of every study on the pooled OR by omitting an individual study at a time. The exclusion of any study did not alter the corresponding pooled OR; *P*-values demonstrated nonsignificant values ranging from 0.15 to 0.99.

## Discussion

This meta-analysis, robustly estimated associations between gene polymorphisms in *TPH1* gene and the risk of suicide behavior. Considerable evidence has shown that the *TPH1* gene is a possible candidate involved in the etiology of suicide. Although one meta-analyses (10) has been conducted in the past 7 years evaluating the relationship between the *TPH1* gene polymorphisms and SB, its findings were inconclusive. Hence, to resolve inconsistencies and to decrease heterogeneity, we performed an updated meta-analysis. Regarding the essential role of genetic factors in the pathogenesis of suicide behavior, we categorized our results according to ethnicity and diagnostic.

First, the pooled analyses results showed that the A218C polymorphism was significantly associated with the risk of SB. The A218C polymorphism was found to be associated with SB in Koreans and Caucasian populations; moreover, A218C has been associated with anger related traits (16, 21, 48). Subgroup analysis based on ethnicity rejected any association between A218C polymorphisms and the SB risk in Asian populations; nonetheless, a significant association between A218C polymorphism and SB susceptibility was detected in European populations. Many reasons might contribute to the conflicting results. First of all, environmental factors that individuals are exposed and different genetic backgrounds, which may have effects on suicide risk. Considering the polygenic effect on psychiatric disorders, the genetic factors that have impacted on the original diseases could share their contributions to suicide as well. In future, large number of case–control studies could provide more evidence for the role of this polymorphism with respect to susceptibility to SB. These findings are consistent with the results of Ono et al. (20), Liu et al. (35) and Abbar et al. (22).

The A218C polymorphism has also been associated with an increased risk of suicide attempt; nonetheless, a protective role was observed in individuals who completed suicide. This polymorphism is localized at intron 7, a site that is a potential GATA transcription factor-binding site. The GATA transcription binding factors allow the initiation of transcription (49). The A218C polymorphism could affect the transcription level of *TPH1*. The TPH is an initial enzyme in the TRYCATs pathway, and its low expression may lead to stopping the pathway.

The tryptophan (TRY) metabolism has two large pathways: the methoxyindole pathway and the kynurenine pathway. Regarding the available TRYs in the body, approximately 1~5% are synthesized as serotonin through the methoxyindole pathway and 95~99% of TRYs are metabolized through the kynurenine pathway and form tryptophan catabolites (TRYCATs) (50, 51). These TRYCATs are important metabolites that may contribute to the pathophysiology of psychiatric disorders such as anxiety and depression (52). TRYCATs potentiate or antagonize relationships with various neurotransmission systems (53). TRYCATs could influence SB by directly contributing to neuroprotective-neurodegenerative changes in the brain. Activation of the TRYCAT pathway leads to the production of a range of neuroactive, neuroprotective and neurotoxic TRYCATs. For example, quinolinic acid act as potent neurotoxin which inhibit ATP production by mitochondria, provoke disrupt neuron glial communication, induce apoptosis of glial cells and directly damage neurons. Other TRYCATS also possess neurotoxic or neuroprotective properties via pro-oxidant and antioxidant effects (54, 55).

Moreover, this polymorphism could also influence individual differences in anger-related personality traits. Individuals carrying the AA genotype have a reduced capacity of controlling anger expression when they experience this emotion. Conversely, the alternative genotypes (AC and CC) can be considered as “protective” against the tendency to lose control when experiencing anger (39). Therefore, we suggest that suicide attempt and completed suicide could be under genetic control and regulated through capacity to control anger.

The observed differences in susceptibility, between suicide attempt and completed suicide, are likely due to the overall genetic background that modifies the SB prone risk factors. Moreover, this discrepancy in SB risk may be explained by daily lifestyle, geographic climate, dietary habits, ethnic diversity and so on. However, these results should be illustrated prudently and need further confirmation by more trials.

Second, A779C and A6526G polymorphisms were not associated with the risk of suicide behavior. Still, we cannot ignore the interaction between *TPH1* and other genes on SB susceptibility, such as *TPH2*. Therefore, it is necessary to systematically screen for functional variants within the *TPH1* gene and other related genes in the SB pathogenesis.

In González-Castro (10) meta-analysis in 2014, our results indicated that the A218C polymorphism was associated with SB, which is consistent with the present study. In comparison with the previous meta-analyses, some advantages of the current study should be addressed. Our study updated the data on *TPH1* polymorphism and the risk of SB and analyzed the role A6526G polymorphism on suicide behavior for the first time. Methodological issues were well explored (e.g., publication bias, sensitivity analysis, heterogeneity analysis) in the present work. Last but not least, the present work was carried out with five genetic models and sub-analyses by population and diagnostic subgroups were performed. Suicide attempt and completed suicide were not analyzed in previous meta-analyses.

The current meta-analysis also has some limitations. First, only articles published in English-language journals were included. Second, inter-gene and gene–environment interactions might also influence the accuracy of our outcomes. A lack of the original data restricted further evaluations of the potential inter-gene and gene–environment interactions. Related to, we recognized as a limitation that considerable percent of the studies were performed in some specific population (e.g. Croatian or German), this possible overlap of clinic center and sample population could introduce bias in the outcome. Therefore, the findings should be taken with caution. Finally, in the suicide ideation group, the number of relevant original documents was limited, there was not enough data to identify the relationship between *TPH1* and suicide ideation. Moreover, the inclusion of the studies that dealt with only ideation may have increased the heterogeneity of the analyzed data.

## Conclusion

The current meta-analysis gives a comprehensive analysis of the available information for the association between the *TPH1* polymorphisms and suicide behavior. This meta-analysis revealed a significant association between the A218C polymorphism of *TPH1* gene and SB. However, neither in overall population nor in subgroup analysis was found a significant association between A779C and A6526G polymorphisms and SB susceptibility. Therefore, the A218C polymorphism could be considered as one possible risk factor of SB. Further studies that investigate the relative contribution of *TPH1* polymorphisms and the mechanisms by which they affect the pathogenesis of SB are required to corroborate these findings.

## Data availability statement

The raw data supporting the conclusions of this article will be made available by the authors, without undue reservation.

## Author contributions

Conceptualization, investigation, methodology, validation, visualization, roles/writing—original draft, and writing—review and editing: YH-D, TG-C, CT-Z, RC-A, ML-N, MR-M, and AG-M. Data curation: YH-D, TG-C, CT-Z, and RC-A. Formal analysis and resources: TG-C, CT-Z, ML-N, and AG-M. Funding acquisition: YH-D, TG-C, CT-Z, and ML-N. Project administration: YH-D, TG-C, and CT-Z. Software: YH-D, TG-C, and CT-Z. Supervision: CT-Z, RC-A, ML-N, MR-M, and AG-M. All authors contributed to the article and approved the submitted version.

## Conflict of interest

The authors declare that the research was conducted in the absence of any commercial or financial relationships that could be construed as a potential conflict of interest.

## Publisher's note

All claims expressed in this article are solely those of the authors and do not necessarily represent those of their affiliated organizations, or those of the publisher, the editors and the reviewers. Any product that may be evaluated in this article, or claim that may be made by its manufacturer, is not guaranteed or endorsed by the publisher.
